# Treatment strategy for lymph node metastasis of hepatocellular carcinoma using an ICG navigation system: a case report

**DOI:** 10.1186/s40792-023-01790-w

**Published:** 2023-12-04

**Authors:** Shunsuke Furukawa, Takao Ide, Kotaro Ito, Tomokazu Tanaka, Hirokazu Noshiro

**Affiliations:** Department of Surgery, Saga University Faculty of Medicine, 5-1-1 Nabeshima, Saga, 849-8501 Japan

**Keywords:** Hepatocellular carcinoma, lymph node metastasis, Indocyanine green, Navigation surgery

## Abstract

**Background:**

Since indocyanine green (ICG) accumulates selectively in hepatocellular carcinoma (HCC) cells, it can be used to detect metastatic lesions. Lymph node metastasis of HCC is rarely observed, both simultaneously and metachronously. Therefore, it is sometimes difficult to identify metachronous lymph nodes during salvage surgery because of prior surgery. Herein, we report a case in which lymph node metastasis of HCC was successfully resected using an ICG navigation system.

**Case presentation:**

The patient was a 62-year-old man who had undergone radical liver resection for HCC 8 years ago. During surveillance, contrast-enhanced computed tomography (CT) revealed a mass in the hepatic hilum. Various diagnostic modalities suggested that the patient had a solitary metastatic lymph node of HCC, and extirpation of the tumor was planned. Intraoperative ICG fluorescence imaging allowed surgeons to clearly identify the target lesion. Histopathologically, the tumor was confirmed to be a lymph node metastasis of HCC. The patient’s postoperative course was uneventful, and he remains alive without recurrence 2 years after the second surgery.

**Conclusion:**

Intraoperative navigation surgery by ICG fluorescence imaging was useful for the safe resection of extrahepatic metastasis of HCC in a complicated situation.

**Supplementary Information:**

The online version contains supplementary material available at 10.1186/s40792-023-01790-w.

## Background

Indocyanine green (ICG), which emits fluorescence upon illumination with near-infrared light, is used to evaluate liver function and surgical navigation [1]. Several studies have demonstrated the usefulness of ICG in the intraoperative detection of hepatocellular carcinoma (HCC) using a near-infrared fluorescence system [2, 3]. In addition, some reports indicate that extrahepatic metastatic lesions from HCC may take up ICG and emit fluorescence when illuminated by near-infrared light [4]. We expect that ICG navigation surgery would be useful for the detection and complete extirpation of extrahepatic lesions, even in a complicated operative field, during repeated surgery. In this report, we describe a case of recurrent HCC in which a metastatic lymph node was successfully removed using intraoperative near-infrared fluorescence imaging with ICG.

## Case presentation

**Fig. 1:**
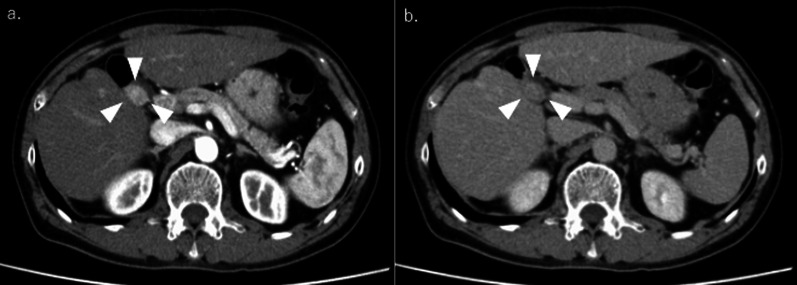
Contrast-enhanced CT. (**a** early phase, **b** delayed phase) showed a mass lesion of 2.5 cm in diameter at the hepatic hilum (white arrow)

**Fig. 2:**
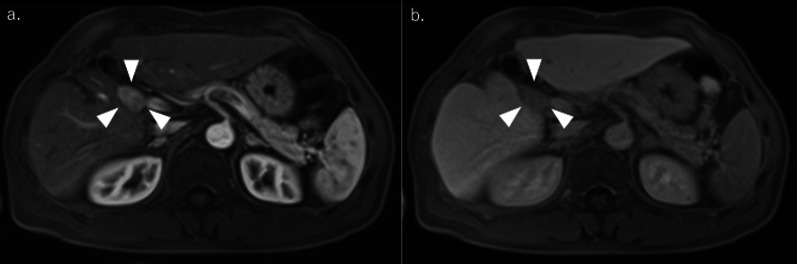
Contrast-enhanced MRI. (**a** early phase, **b** delayed phase) similarly shows a mass lesion at the hepatic hilum (white arrow)

**Fig. 3:**
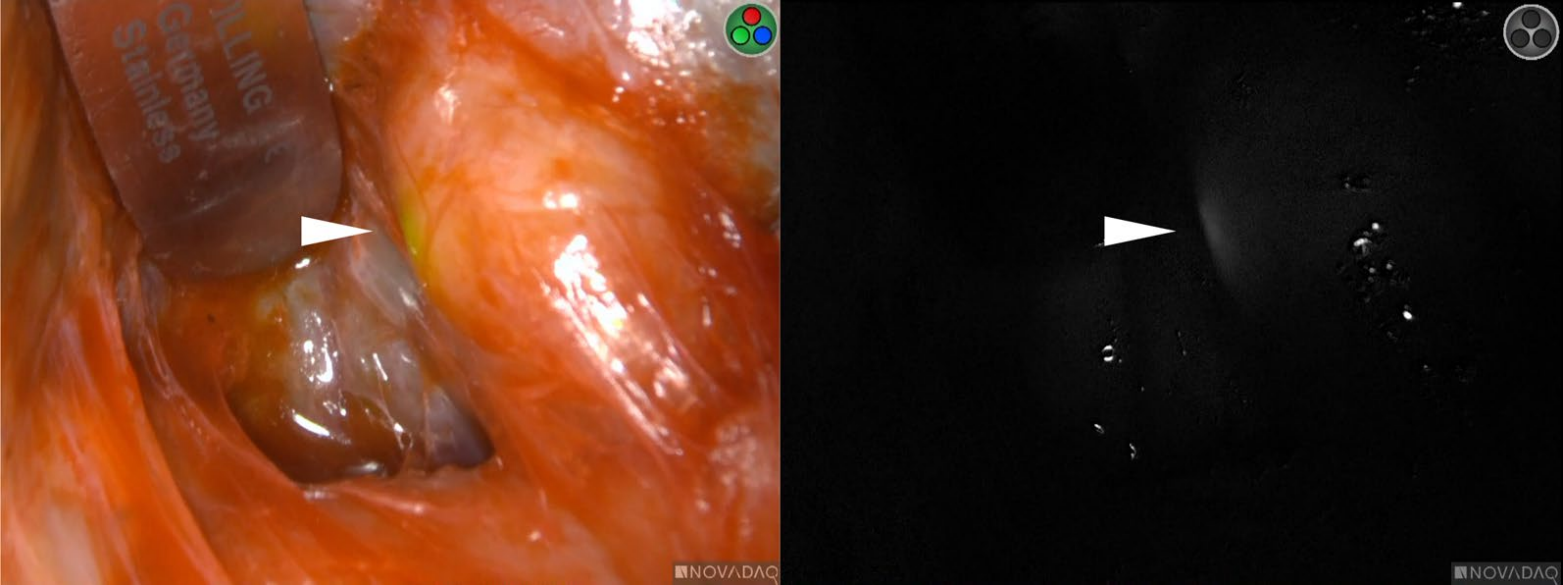
ICG fluorescence. The tumor at the hepatic hilum showed ICG fluorescence (white arrowhead)

**Fig. 4:**
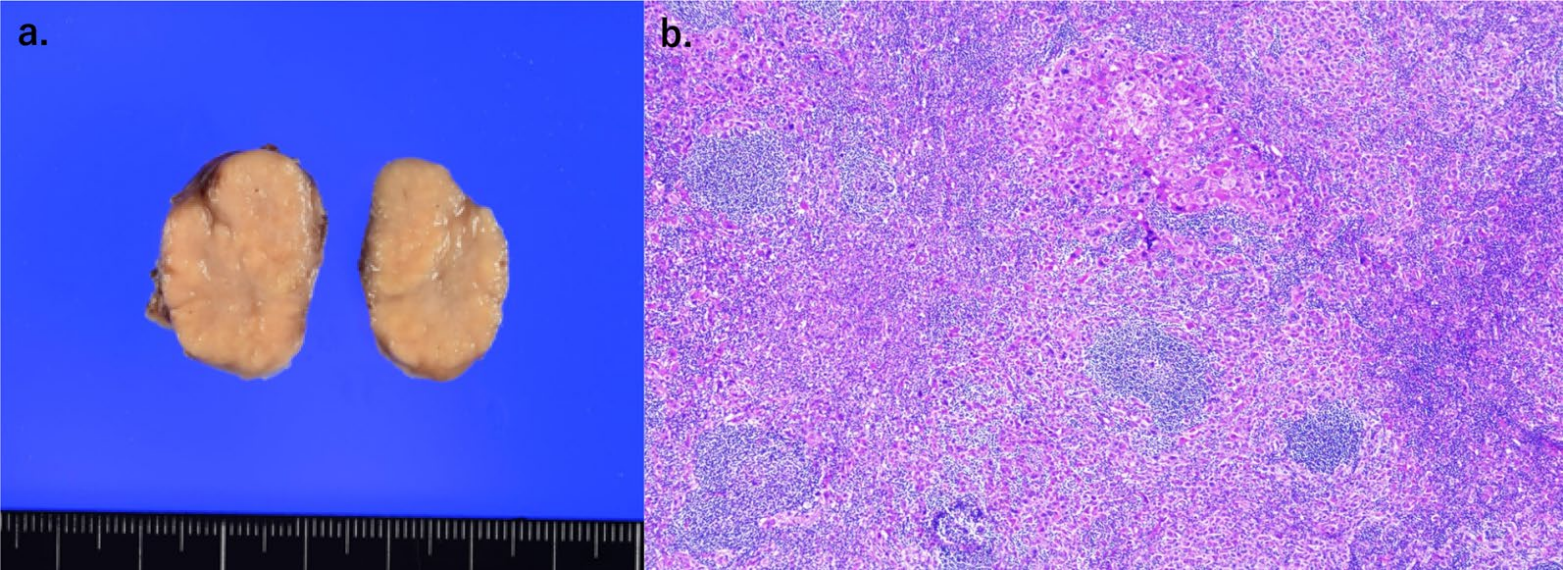
Pathological examination of the tumor. **a** Macroscopic findings of the resected specimens. **b** Hematoxylin and eosin staining of resected specimens revealed that the normal lymph node structure was replaced by moderately differentiated HCC (HE, × 40)

The patient was a 62-year-old male. His relevant medical history included chronic hepatitis C, which was cured with interferon. He had undergone extended right anterior sectionectomy of the liver for cStage II HCC 8 years previously. Hematoxylin and eosin staining of the resected specimen revealed a moderately differentiated HCC of the simple nodular type. During surveillance, contrast-enhanced computed tomography (CT) revealed a mass lesion measuring 2.5 cm in diameter at the hepatic hilum, suggesting lymph node metastasis (Fig. 1). Contrast-enhanced magnetic resonance imaging (MRI) revealed a mass with early staining and gradual washout at the hepatic hilum (Fig. 2). This mass lesion had the same contrast pattern as the initial HCC. The tumor did not involve major vessels or other organs. No distant metastases other than the mass lesions were observed. The patient’s laboratory results were unremarkable. Tumor markers of alpha-feto protein (AFP) and protein induced by vitamin K absence or antagonist-II (PIVKA-II) were also normal (2.2 ng/mL and 13.0 mAU/mL, respectively). The AFP level was within the normal range (4.6 ng/mL); however, PIVKA-II level was elevated (1058 mAU/mL) before the initial surgery. The patient exhibited a well-preserved liver function. Based on the above findings, we diagnosed the patient with solitary metastatic lymph node HCC, and extirpation of the tumor was planned. To detect the location of the tumor in the complicated adhesive surgical field around the hepatic hilum (due to prior surgery), ICG for intraoperative navigation surgery was injected intravenously at a dose of 0.5 mg/kg body weight (total dose 24.5 mg), 3 days before the current surgery. After laparotomy, the severe adhesions were released, and the hepatic hilum was explored. As expected, the tumor showed partial fluorescence under a PINPOINT Endoscopic fluorescence imaging system (Stryker Japan K.K.), a near-infrared light camera system (Fig. 3 and Additional file 1: Video S1). ICG fluorescence from the tumor itself was no longer observed at the hepatic hilum after tumor removal. The operative time was 118 min, and blood loss was minimal. The pathological diagnosis was lymph node metastasis of the HCC. Hematoxylin and eosin staining of the resected specimen revealed that the normal lymph node structure was replaced by a moderately differentiated HCC, which was consistent with the findings of the initial excised specimen (Fig. 4). The patient was discharged on postoperative day 9 without complications. The patient remains alive without recurrence of HCC 2 years after the second surgery.

## Discussion

Extrahepatic recurrence of HCC is rare compared with intrahepatic recurrence after curative liver resection. The most common sites of extrahepatic recurrence are the lungs, bones, lymph nodes, peritoneum, adrenal glands, and the brain. Lymph node recurrence after curative liver resection is found in only 1.6% of the cases [5]. Although the clinical significance of surgical resection of such lymph node metastases remains controversial, the efficacy of local therapy, including surgical resection of extrahepatic lesions, has been reported. Some studies have reported that complete removal of metastatic lymph nodes is clinically beneficial for patients with HCC with resectable or controllable intrahepatic lesions [6, 7]. In addition, survival rates are expected to improve after resection of solitary lymph node metastases [7]. In the present case, the patient had solitary lymph node metastasis and no other recurrent lesions for > 8 years after the initial operation. Thus, in this case, we considered that a survival benefit could be expected from surgery for lymph node recurrence.

ICG binds to Î±-lipoproteins, Î^2^-lipoproteins, albumin, and α2-globulin. Fluorescent imaging using ICG can detect liver cancers through visualization of the disordered biliary excretion of ICG in cancer and noncancerous liver tissues compressed by the tumor. ICG-fluorescence imaging enables highly sensitive identification of tumor location, thereby contributing to accurate liver resection [3]. In contrast, ICG fluorescence has been reported to be more useful in detecting extrahepatic metastasis than in detecting primary HCC and intrahepatic metastasis. ICG remains in tumor cells for a longer time because there is no transbiliary excretion pathway from tumor tissue to non-tumor tissue. In addition, the contrast of ICG fluorescence is enhanced in extrahepatic metastases because of the absence of fluorescence in the background [8]. Previous studies have shown that ICG fluorescence is useful for detecting metastatic lymph nodes in HCC [4, 9, 10]. In fact, Satou et al. revealed that lymph node metastasis could be detected by only faint fluorescence of ICG after dissecting the surrounding tissue, even if it was deeply present in the soft tissue [4]. In agreement with these reports, faint fluorescence of ICG in metastatic lymph nodes was also observed after dissecting the severe adhesions in our case, following accurate identification of the outline of the tumor. Thus, the intraoperative use of ICG has the potential to detect small extrahepatic metastases that cannot be detected on preoperative imaging despite the small number of reports on surgical treatment for metastatic lesions.

The ICG fluorescence pattern depends on the differentiation level of HCC [11]. Well-differentiated HCC shows intense and homogeneous fluorescence, moderately differentiated HCC shows partial fluorescence, and poorly differentiated HCC and liver metastases show a fluorescent halo corresponding to peritumor fluorescence. Thus, ICG is less likely to accumulate in poorly differentiated HCC than in the well-differentiated ones. In previous studies, ICG was injected intravenously at a dose of 0.5 mg/kg body weight, 3 days (1–5 days) before surgery to investigate lymph node metastasis, as well as pulmonary, adrenal, and peritoneal metastasis of HCC. Consequently, these reports showed the potential advantages of ICG for the intraoperative detection of extrahepatic metastasis [4, 12, 13]. In our case, ICG was injected intravenously before surgery at the same dose used for the evaluation of liver function, and the final pathological diagnosis was moderately differentiated HCC. ICG fluorescence, which had a partial accumulation similar to that described in previous reports, was detected in the metastatic lymph nodes. However, the optimal timing and dose of ICG administration remains to be determined [14]. To our knowledge, there have been only three reports of surgical treatment with ICG for lymph node metastasis in HCC [4, 9, 10]. Further studies are required to clarify the relationship between ICG uptake and lymph node metastasis in HCC. This would contribute to the establishment of a surgical treatment strategy for extrahepatic HCC metastasis.

## Conclusion

Intraoperative ICG fluorescence imaging is useful for safe and complete removal of lymph nodes involved in HCC.

### Supplementary Information

**Additional file 1: Video S1.** ICG fluorescence was observed in the tumor after dissection of severe adhesions.

## Data Availability

Not applicable.

## Abbreviations

ICG: Indocyanine green
HCC: Hepatocellular carcinoma
CT: Computed tomography
MRI: Magnetic resonance imaging
AFP: Alpha-feto protein
PIVKA-II: Protein induced by vitamin K absence or antagonist-II
